# ASO Author Reflections: Neuron Projection Guidance—A transcriptomic perspective on tumor-induced neural recruitment in breast cancer

**DOI:** 10.1245/s10434-026-20049-w

**Published:** 2026-08-10

**Authors:** Kei Kawashima, Kohei Chida, Masanori Oshi, Akimitsu Yamada, Kazutaka Narui, Shipra Gandhi, John M. L. Ebos, Scott I. Abrams, Arin Bhattacharjee, Itaru Endo, Kazuaki Takabe

**Affiliations:** 1 Department of Surgical Oncology, Roswell Park Comprehensive Cancer Center, Buffalo, NY USA; 2 Department of Gastroenterological Surgery, Yokohama City University Graduate School of Medicine, Kanagawa, Japan; 3 Department of Breast and Thyroid Surgery, Yokohama City University Medical Center, Yokohama City, Kanagawa Japan; 4 Department of Medicine, Winship Cancer Institute of Emory University, Atlanta, GA USA; 5 Department of Cancer Genetics and Genomics, Roswell Park Comprehensive Cancer Center, Buffalo, NY USA; 6 Department of Immunology, Roswell Park Comprehensive Cancer Center, Buffalo, NY USA; 7 Department of Pharmacology and Toxicology, University at Buffalo Jacobs School of Medicine and Biomedical Sciences, The State University of New York, Buffalo, NY USA; 8 Department of Surgery, University at Buffalo Jacobs School of Medicine and Biomedical Sciences, The State University of New York, Buffalo, NY USA; 9 Division of Digestive and General Surgery, Niigata University Graduate School of Medical and Dental Sciences, Niigata, Japan; 10 Department of Breast Surgery and Oncology, Tokyo Medical University, Tokyo, Japan; 11 Department of Breast Surgery, Fukushima Medical University, Fukushima, Japan

## Past

Intratumoral nerves are increasingly recognized as a key component of the tumor microenvironment (TME) and are associated with aggressive tumor biology and poorer outcomes.^1^ However, quantification of nerve density in a bulk tumor using histological^2^ or transcriptome approaches is challenging because neuronal cell bodies are located outside of the tumor.^3^ It was reported that tumors recruit neural projections into the TME through neurotrophic factors and axon guidance molecules,^4^ a process termed neuron projection guidance (NPG). Hence, we hypothesized that NPG activity, which is expected to be associated with neural abundance, could help characterize neural-associated biology in breast cancer.

## Present

NPG activity was assessed using an NPG score comprising 244 signature genes across multiple breast cancer cohorts.^5^ NPG score was indeed associated with axonogenesis-related gene expression. NPG-high tumors were associated with features of reduced proliferative activity and lower mutational burden and neoantigen load. Consistent with these findings, NPG-high tumors exhibited an immune-cold TME characterized by low infiltration of immune effector cells, decreased features of cytolytic activity, and suppression of immune-related pathways. In addition, the NPG score was higher in patients who did not experience pathological complete response after neoadjuvant chemotherapy. An elevated NPG score was also associated with worse survival outcomes. These associations were particularly significant in triple-negative breast cancer.

## Future

NPG activity may serve as an indicator reflecting a nerve-rich TME, which may form distinct phenotypic characteristics within more aggressive breast cancer. As tumor–nerve interactions are increasingly recognized across multiple cancer types, focusing on the NPG process may provide a new framework for investigating nerve-associated cancer biology. However, this score captures the overall process of neural recruitment rather than nerve fibers themselves, and the observational nature of this study precludes definitive mechanistic conclusions. Further experimental validation is needed to clarify the underlying molecular mechanisms.
